# Efficacy and safety of low‐dose atorvastatin plus ezetimibe for primary hypercholesterolemia: A randomized, double‐blind, multicenter phase 3 trial

**DOI:** 10.1002/lipd.12442

**Published:** 2025-04-18

**Authors:** Tae Oh Kim, Kyounghoon Lee, Jin‐Man Cho, Hyuck‐Jun Yoon, Tae‐Ho Park, Jung Hyun Choi, Jung‐Won Suh, Seok‐Yeon Kim, Hong‐Seok Lim, Jong‐Seon Park, Deok‐Kyu Cho, Gyung‐Min Park, Sung‐Gyun Ahn, Sanghoon Shin, Sung Uk Kwon, Dae‐Hyeok Kim, Sang‐Rok Lee, Jung‐Hoon Sung, Hwan‐Cheol Park, Seung‐Whan Lee

**Affiliations:** ^1^ Department of Cardiology University of Ulsan College of Medicine, Asan Medical Center Seoul Korea; ^2^ Department of Cardiology, Gil Medical Center Gachon University of Medicine and Science Incheon Republic of Korea; ^3^ Department of Cardiology Kyung Hee University Hospital at Gangdong Seoul Republic of Korea; ^4^ Department of Cardiology, Dongsan Hospital Keimyung University College of Medicine Daegu Republic of Korea; ^5^ Division of Cardiology, Department of Internal Medicine Dong‐A University College of Medicine Busan Republic of Korea; ^6^ Division of Cardiology, Department of Internal Medicine Pusan National University Hospital Busan Republic of Korea; ^7^ Division of Cardiology, Department of Internal Medicine Seoul National University Bundang Hospital, Seoul National University College of Medicine Seongnam Republic of Korea; ^8^ Department of Cardiology Cardiovascular Center, Seoul Medical Center Seoul Republic of Korea; ^9^ Department of Cardiology Ajou University School of Medicine Suwon Republic of Korea; ^10^ Division of Cardiology, Department of Internal Medicine Yeungnam University Hospital Daegu Republic of Korea; ^11^ Division of Cardiology Yonsei University College of Medicine and Cardiovascular Center, Yongin Severance Hospital Yongin Republic of Korea; ^12^ Department of Cardiology Ulsan University Hospital, University of Ulsan College of Medicine Ulsan Republic of Korea; ^13^ Department of Internal Medicine, Wonju Severance Christian Hospital Yonsei University Wonju College of Medicine Wonju Republic of Korea; ^14^ Department of Cardiology Ewha Womans University Seoul Hospital Seoul Republic of Korea; ^15^ Department of Cardiology Ilsan Paik Hospital, Inje University, College of Medicine Goyang Republic of Korea; ^16^ Division of Cardiology Inha University Hospital Incheon Republic of Korea; ^17^ Division of Cardiology Chonbuk National University Hospital and Chonbuk School of Medicine Jeonju Republic of Korea; ^18^ Division of Cardiology, CHA Bundang Medical Center CHA University Seongnam Republic of Korea; ^19^ Division of Cardiology Hanyang University Guri Hospital Guri Republic of Korea

**Keywords:** atorvastatin, clinical trial, ezetimibe, hypercholesterolemia, LDL, lipoprotein, phase III

## Abstract

Studies have suggested that low‐dose statin monotherapy may be insufficient for target LDL‐C levels. In this randomized, double‐blind, multicenter phase 3 trial, we evaluated the efficacy of combined ezetimibe and low‐dose atorvastatin in 222 Korean patients with primary hypercholesterolemia. Participants received either 10‐mg ezetimibe/5‐mg atorvastatin (EZE10/ATV5), 10‐mg ezetimibe (EZE10), 5‐mg atorvastatin (ATV5), or 10‐mg atorvastatin (ATV10). At 8 weeks, EZE10/ATV5 achieved the greatest LDL‐C reduction (−44.8%) compared with EZE10 (−12.7%, *p* < 0.0001), ATV5 (−27.3%, *p* < 0.0001), and ATV10 (−32.0%, *p* = 0.0012). The combination therapy showed the highest LDL‐C goal achievement rate (41.1% vs. EZE10 8.9%, *p* < 0.0001; ATV5 10.9%, *p* < 0.0001; ATV10 27.3%, *p* = 0.0342), particularly in moderate to high‐risk patients. Additionally, EZE10/ATV5 had the lowest adverse events among all groups (6.9% vs. 15.0%, 12.3%, and 27.6%, *p* = 0.017), with most events being mild. These findings suggest that the combination of ezetimibe and low‐dose atorvastatin provides superior lipid‐lowering efficacy with an improved safety profile, offering an effective treatment for primary hypercholesterolemia in Korean patients.

## Graphical abstract

Low‐dose atorvastatin combined with ezetimibe achieves superior LDL‐C reduction (44.8%) compared with equivalent or higher‐dose statin monotherapy in Korean patients, presenting a more effective and safer therapeutic strategy potentially applicable to broader Asian populations.

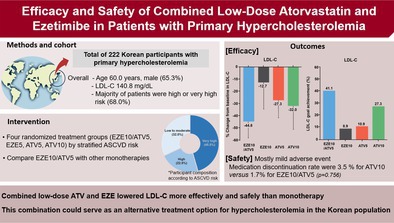

**Abbreviations**
AEs: adverse events
ALT: alanine aminotransferase
ANCOVA: analysis of covariance
Apo A1: apolipoprotein A1
Apo B: apolipoprotein B
AST: aspartate aminotransferase
ATV5: atorvastatin 5 mg
ATV10: atorvastatin 10 mg
CK: creatinine kinase
CVD: cardiovascular disease
DM: diabetes mellitus
EAS: European atherosclerosis society
ESC: European society of cardiology
EZE10: ezetimibe 10 mg
HDL‐C: high‐density lipoprotein cholesterol
HTN: hypertension
LDL‐C: low‐density lipoprotein cholesterol
SD: standard deviation
TC: total cholesterol
TAG: triacylglycerol

## INTRODUCTION

Cardiovascular disease (CVD) is the leading cause of morbidity and mortality worldwide, despite continuous improvements in diagnosis, therapeutics, and risk factor control (Roth et al., 2020). Hypercholesterolemia is a major modifiable risk factor for cardiovascular events, and actively lowering low‐density lipoprotein cholesterol (LDL‐C) is essential for both primary and secondary prevention of atherosclerotic CVD (Magnussen et al., 2023). Statins are well established as first‐line pharmacotherapy for hypercholesterolemia, with guideline‐recommended intensities necessary to achieve target LDL‐C levels based on the cardiovascular risk of the patient (Grundy et al., 2019; Mach et al., 2020). However, these guidelines, often derived from Western populations, may not be appropriate for Asians, who tend to achieve target LDL levels with lower statin doses due to pharmacogenetic differences (Naito et al., 2017). High‐intensity statin therapies, as typically endorsed in Western guidelines, potentially pose an increased risk of adverse effects without additional benefits in this population.

Recent evidence suggests that combining lower doses of statins with ezetimibe can further reduce side effects while effectively lowering LDL levels (Kim et al., 2022). This combination therapy achieves a magnitude of LDL‐C reduction similar to that of higher statin doses alone, but with fewer adverse effects. Consequently, the addition of ezetimibe to a low‐intensity statin regimen is particularly beneficial for Asians, enhancing lipid control and treatment tolerability. This study aimed to evaluate the efficacy and safety of combining low‐dose atorvastatin, a statin with well‐established evidence, with ezetimibe. This approach aligns with broader efforts to optimize cardiovascular risk management through population‐specific therapeutic regimens, particularly for Asian populations where lower statin doses may achieve optimal outcomes.

## METHODS

### Study design

A randomized, double‐blind, multicenter, therapeutic confirmatory phase 3 clinical trial was conducted at 19 sites in Korea from November 2022 to July 2023. The study was divided into two periods: a 4‐week placebo run‐in period and an 8‐week double‐blind treatment period (Figure S1). Any previous lipid‐lowering drugs were discontinued during the 4‐week run‐in period. After the run‐in period, the participants were randomized 1:1:1:1 to ezetimibe 10 mg and atorvastatin 5 mg (EZE10/ATV5), ezetimibe 10 mg (EZE10), atorvastatin 5 mg (ATV5), and atorvastatin 10 mg (ATV10) for 8 weeks. To maintain strict double‐blind conditions, all treatment arms received three tablets per day—comprising both active drug and matching placebo tablets as needed—so that the total tablet count and appearance were indistinguishable among the groups. All investigational products were identical in appearance and weight. Medication was assigned according to a centralized randomization list, which was inaccessible to investigators and patients throughout the trial. This prevented subjects, investigators, and study monitors from identifying any particular treatment arm or inferring a “preferred” intervention. The randomization was stratified according to the cardiovascular risk categories of the European Society of Cardiology (ESC) and European Atherosclerosis Society (EAS) guidelines (2019) (Table S1). During the run‐in and treatment periods, participants followed therapeutic lifestyle changes. Demographic data were collected prior to the run‐in period, while laboratory measurements—used to confirm lipid levels and eligibility—were performed at the end of the run‐in period and immediately before randomization. The inclusion criteria were primary hypercholesterolemia, age ≥ 19 years, LDL‐C levels ≤250 mg/dL, and triacylglycerol (TAG) levels <500 mg/dL. The exclusion criteria were secondary hypercholesterolemia, liver disease, renal disease, type 1 diabetes mellitus, uncontrolled type 2 diabetes mellitus, uncontrolled hypertension, uncontrolled hyper‐ or hypothyroidism, active cardiovascular disease, active cerebrovascular disease, impaired drug absorption, a history of myopathy, malignancy within the last 5 years, hypersensitivity reaction to atorvastatin or ezetimibe, drug or alcohol abuse within the last 24 weeks, treatment with fibrates within the last 2 weeks, or other investigational drugs within the last 8 weeks, pregnancy, or breastfeeding.

### Endpoints and sample size

The primary endpoint was the percentage change in LDL‐C levels from baseline at week 8. The secondary endpoints were the percentage change in LDL‐C levels from baseline at week 4, the percentage change in lipid parameters (total cholesterol [TC], TAG, high‐density lipoprotein cholesterol [HDL‐C], non‐HDL‐C, apolipoprotein B [Apo B], and apolipoprotein A1 [Apo A1]) from baseline at weeks 4 and 8, and the achievement rate of target LDL‐C levels at week 8. Target LDL‐C levels were defined according to the ESC/EAS guidelines (2019) based on cardiovascular risk categories: <70 mg/dL for very high‐risk, <100 mg/dL for high‐risk, and < 116 mg/dL for moderate‐risk patients. LDL‐C, TC, TAG, and HDL‐C were analyzed using a Cobas c502 (Hitachi, Japan) at a central laboratory, and Apo B and Apo A1 were analyzed using a Cobas c051 (Roche, Japan) at a central laboratory. Safety was evaluated through adverse events (AEs), laboratory tests, physical examination, vital signs assessments, and weight measurements.

The sample was 228 participants (57 participants per group). This provided 85% power to detect a difference of 14.3% in the LDL‐C percentage change from baseline at week 8 among the groups. This calculation was based on a two‐sided test at an alpha of 0.05, a common standard deviation of 24.0%, and a dropout rate of 10%.

### Statistical analysis

The baseline characteristics were compared between the groups. A descriptive analysis was performed by presenting the data as means ± standard deviations or numbers (proportion). Continuous variables were compared with a one‐way ANOVA test (for normally distributed data) or a Kruskal–Wallis test (for non‐normally distributed data), and categorical variables were compared between groups with the chi‐square test or Fisher's exact test.

For primary and secondary endpoints, the continuous variables (lipid parameters) were evaluated by analysis of covariance with relevant baseline measurements and cardiovascular risk categories as covariates. The categorical variables (LDL‐C level target achievement rates) were evaluated by the Cochran–Mantel–Haenszel test. For safety analysis, the Chi‐square test was used when expected frequencies were ≥5 in all cells, and Fisher's exact test was used when any expected frequency was <5. The missing values were imputed using the last observation carried forward. This study aimed to demonstrate that EZE10/ATV5 is more effective than EZE10 and ATV5 in reducing LDL‐C levels. ATV10, a standard moderate‐intensity statin, served as the benchmark for evaluating the efficacy of EZE10/ATV5 in lowering LDL‐C.

The full analysis set comprised patients who received at least one dose of the investigational drug and had at least one primary endpoint assessment post‐administration. The safety analysis set included all participants who received at least one dose of the investigational drug. The full and safety analyses were performed for the intention‐to‐treat population. Two‐sided tests were performed at a significance level of 0.05 for all statistical analyses. All statistical analyses were performed with SAS version 9.4 (SAS Institute, Inc., Cary, North Carolina, USA).

## RESULTS

### Baseline characteristics

**FIGURE 1:**
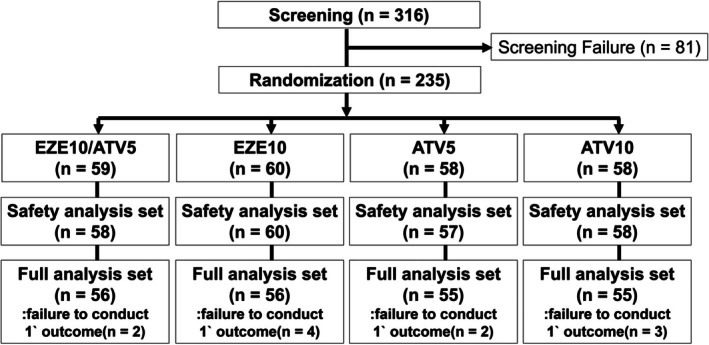
Study flow diagram. This figure shows the flow of participants in this study, which evaluated different doses and combinations of ezetimibe and atorvastatin. Starting with 316 individuals, 81 were excluded at screening due to not meeting inclusion criteria or meeting exclusion criteria. The remaining 235 participants were divided into four groups: EZE10/ATV5, EZE10, ATV5, and ATV10. The numbers of participants who completed the safety and efficacy evaluations are shown for each group. ATV5, atorvastatin 5 mg monotherapy; ATV10, atorvastatin 10 mg monotherapy; EZE10, ezetimibe 10 mg monotherapy; EZE10/ATV5, combination therapy of ezetimibe 10 mg and atorvastatin 5 mg.

A total of 316 participants were screened, and of the 235 participants who were randomized, 24 (10.2%) did not complete the study, while 211 (89.8%) completed the study (Figure 1). A total of 233 participants (99.2%) were included in the safety analysis set, and 222 participants (94.5%) were included in the full analysis set. In the study, treatment compliance among the randomly assigned patients ranged from 95.2% to 97.7% across the groups, with no significant differences noted (Table S2).

The baseline clinical characteristics of the study population are summarized in Table 1. Overall, there were no significant differences between the groups in demographics, cardiovascular risk factors, and renal function. Despite differences in pre‐run‐in statin use (with EZE10 group having fewer statin‐naïve patients at 26.8% vs. 40.0%–44.6% in other groups), all lipid parameters measured after the 4‐week medication washout period were comparable across groups. The distribution of cardiovascular risk categories was also similar across the groups, indicating that the study cohorts were comparable at baseline.

**TABLE 1:** Baseline clinical characteristics of the study population.

	EZE10/ATV5 (*n* = 56)	EZE10 (*n* = 56)	ATV5 (*n* = 55)	ATV10 (*n* = 55)	*p*‐value
Age (years)	60.8 ± 12.2	59.9 ± 11.8	60.9 ± 10.4	57.6 ± 12.4	0.404
Male	35 (62.5)	38 (67.9)	34 (61.8)	38 (69.1)	0.802
BMI (kg/m^2^)^a^	25.4 ± 3.2	25.1 ± 3.6	24.9 ± 3.6	26.7 ± 4.4	0.199
Hypertension	31 (55.4)	33 (58.9)	33 (60.0)	39 (70.9)	0.370
Diabetes	15 (26.8)	15 (26.8)	19 (34.5)	16 (29.1)	0.783
Current smoking	7 (12.5)	10 (17.9)	11 (20.0)	13 (23.6)	0.251
Coronary artery disease	25 (44.6)	25 (44.6)	29 (52.7%)	27 (49.1)	0.797
Duration of hyperlipidemia (years)	6.4 ± 6.6	5.9 ± 5.3)	5.1 ± 4.7	5.8 ± 5.0	0.945
Premature family history of CAD^b^	2 (3.57)	5 (8.9)	‐	2 (3.6)	0.142
Pre‐run‐in lipid‐lowering therapies^c^					
Statin‐Naïve	25 (44.6%)	15 (26.8%)	22 (40.0%)	22 (40.0%)	0.473
On statin therapy	31 (55.4%)	41 (73.2%)	33 (60.0%)	33 (60.0%)	
Low‐intensity statin	4 (7.1%)	4 (7.1%)	0 (0%)	3 (5.5%)	
Moderate~high‐intensity statin	27 (48.2%)	37 (66.0%)	33 (60.0%)	30 (54.5%)	
Combination with ezetimibe	10 (17.9%)	15 (26.8%)	12 (21.8%)	12 (21.8%)	0.728
Estimated GFR	88.9 ± 16.1	88.0 ± 14.8	90.3 ± 14.8	89.3 ± 16.6	0.888
Total cholesterol (mg/dL)^c^	216.6 ± 33.0	211.8 ± 32.7	209.2 ± 30.5	205.4 ± 29.7	0.299
Triacylglycerol (mg/dL)^c^	143.5 (103.0–192.8)	139.5 (86.5–225.5)	136.0 (99.0–222.0)	142.0 (111.0–191.0)	0.723
HDL‐C (mg/dL)^c^	50.7 ± 14.1	51.7 ± 13.2	48.4 ± 14.5	49.6 ± 11.7	0.599
LDL‐C (mg/dL)^c^	143.8 ± 25.8	137.4 ± 26.9	137.3 ± 25.0	133.8 ± 27.4	0.244
Apo B (mg/dL)^c^	119.8 ± 20.6	116.2 ± 22.6	119.0 ± 23.1	115.5 ± 21.6	0.691
Risk category^d^					
Very high	25 (44.6)	24 (42.9)	28 (50.9)	24 (43.6)	0.999
High	13 (23.2)	13 (23.2)	11 (20.0)	13 (23.6)	
Low to moderate	18 (32.1)	19 (33.9)	16 (29.1)	18 (32.7)	

Abbreviations: Apo B, apolipoprotein B; ATV5, atorvastatin 5 mg monotherapy; ATV10, atorvastatin 10 mg monotherapy; CAD, coronary artery disease; EZE10, ezetimibe 10 mg monotherapy; EZE10/ATV5, combination therapy of ezetimibe 10 mg and atorvastatin 5 mg; GFR, glomerular filtration rate; HDL‐C, high‐density lipoprotein cholesterol; LDL‐C, low‐density lipoprotein cholesterol.

^a^ Body mass index (BMI) is calculated as weight in kilograms divided by height in meters squared (kg/m^2^).

^b^ Premature family history of CAD: CAD in a male first‐degree relative <55 years or a female first‐degree relative <65 years.

^c^ Demographic data and lipid‐lowering therapies were collected before the run‐in period, while lipid parameters were measured after the 4‐week run‐in period, immediately before randomization.

^d^ The 2019 ESC/ESA Guidelines categorize 10‐year risk based on SCORE2 into low, moderate, high, and very high.

### Primary and secondary endpoints

**FIGURE 2:**
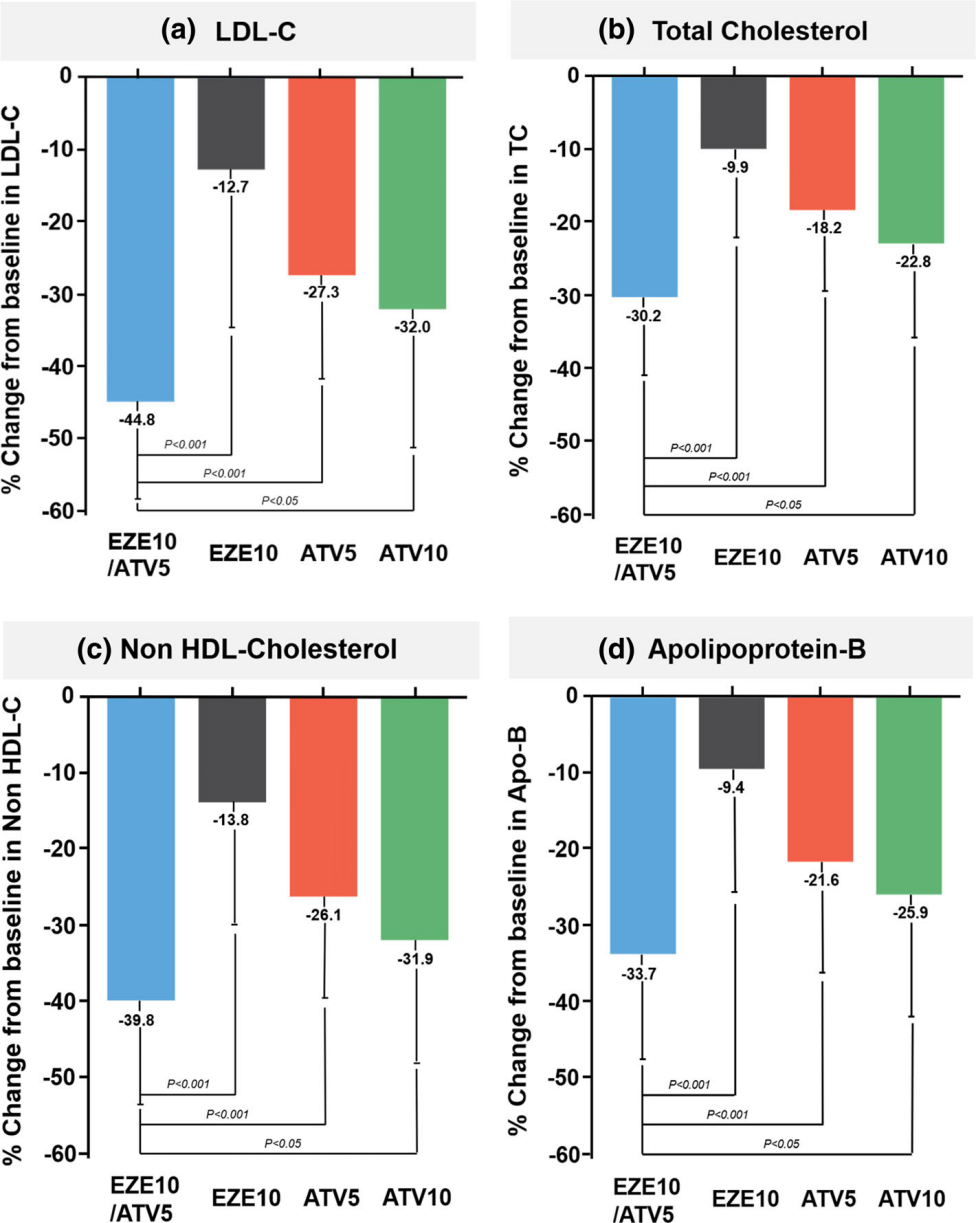
Changes in lipid levels by treatment group. This figure illustrates the percentage change from baseline in lipid levels in different ezetimibe and atorvastatin treatment groups: (a) LDL‐C, (b) TC, (c) non‐HDL‐C, and (d) Apo B. Each bar represents the mean percent change (± standard deviation), with *p*‐values indicating statistical significance between groups, highlighting the effect of each treatment on lipid levels. Apo B, apolipoprotein B; ATV, atorvastatin; EZE, ezetimibe; LDL‐C, low‐density lipoprotein cholesterol; non‐HDL‐C, non‐high‐density lipoprotein cholesterol; TC, total cholesterol.

The reduction in LDL‐C levels at week 8 was significantly greater in the EZE10/ATV5 group (−44.8 ± 13.5%) than in the EZE10 group (−12.7 ± 21.9%, *p* < 0.001), ATV5 group (−27.3 ± 15.7%, *p* < 0.001), and ATV10 group (−32.0 ± 19.7%, *p* < 0.05) (Figure 2a). The LDL‐C lowering effect appeared to be fully achieved by week 4, with stable levels maintained through week 8 (Figure S2). While the degree of reduction varied among the different lipid profile components, the EZE10/ATV5 group also showed the most pronounced reductions in lipid parameters (TC, non‐HDL‐C, and Apo B) (Figure 2b–d), with the most substantial reductions observed within the first 4 weeks (Table S3).

### Target achievement rates

**FIGURE 3:**
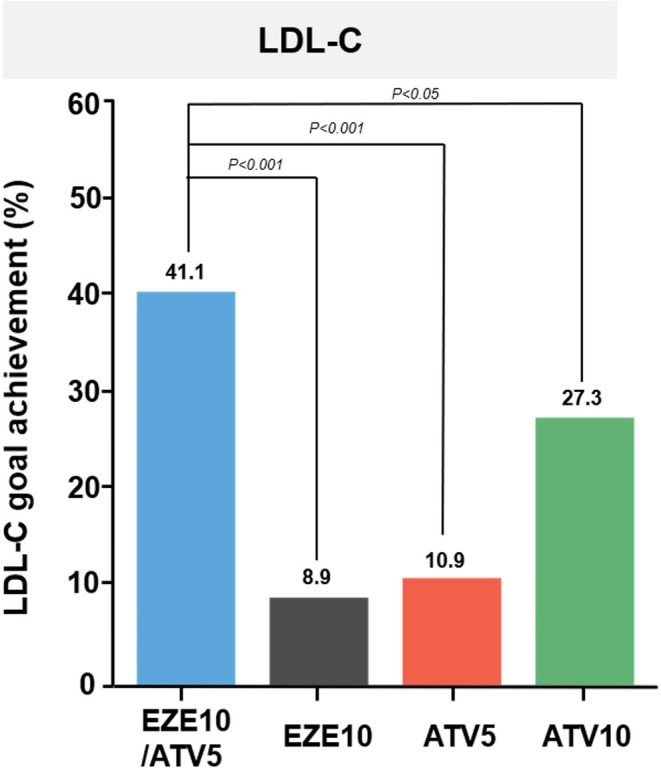
Target LDL‐C achievement by treatment group. Each bar shows the percentage of patients that achieved their risk‐based LDL‐C goals (<55 mg/dL for very high‐risk, <70 mg/dL for high‐risk, <100 mg/dL for moderate‐risk patients, and <116 mg/dL for low‐risk patients) in the different treatment groups. Statistical significance between treatments is indicated by the *p*‐values above the parentheses connecting the bars. ATV5, atorvastatin 5 mg monotherapy; ATV10, atorvastatin 10 mg monotherapy; EZE10, ezetimibe 10 mg monotherapy; EZE10/ATV5, combination therapy of ezetimibe 10 mg and atorvastatin 5 mg.

The proportion of participants who achieved target LDL‐C levels at week 8 was significantly greater in the EZE10/ATV5 group (41.1%) than in the EZE10 group (8.9%, *p* < 0.001), ATV5 group (10.9%, *p* < 0.001), and ATV10 group (27.3%, *p* < 0.05) (Figure 3).

**FIGURE 4:**
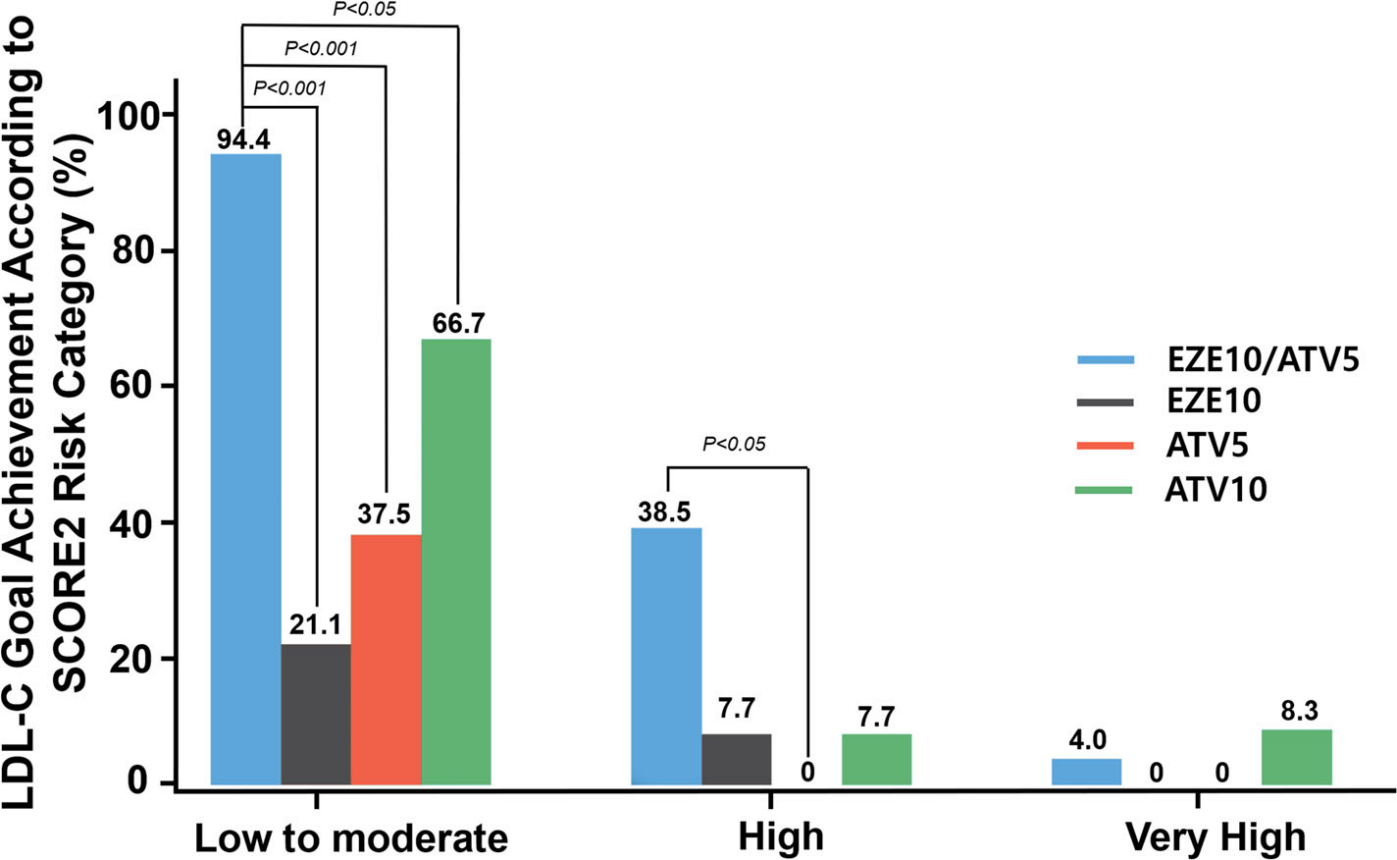
Target LDL‐C achievement by SCORE2 risk and treatment group. Each bar chart shows target LDL‐C achievement percentages for patients categorized by SCORE2 risk levels (low to moderate, high, and very high). The treatments are represented by different colored bars: EZE10/ATV5 (blue), EZE10 (black), ATV5 (red), and ATV10 (green). Each bar represents the percentage of patients within each risk category that achieved their LDL‐C goals (<116 mg/dL for low‐risk, <100 mg/dL for moderate‐risk, <70 mg/dL for high‐risk, and <55 mg/dL for very high‐risk patients). Statistical significance is indicated by the *p*‐values above the parentheses comparing treatments. ATV5, atorvastatin 5 mg monotherapy; ATV10, atorvastatin 10 mg monotherapy; EZE10, ezetimibe 10 mg monotherapy; EZE10/ATV5, combination therapy of ezetimibe 10 mg and atorvastatin 5 mg; SCORE2, Systematic COronary Risk Evaluation2.

Analysis by SCORE2 Risk Category showed that in the low to moderate risk category, the EZE10/ATV5 group had 94.4% goal achievement, compared with 21.1% in EZE10, 37.5% in ATV5, and 66.7% in ATV10 (*p* < 0.05 for all comparisons) (Figure 4). In high‐risk patients, goal achievement rates were 38.5% for EZE10/ATV5, 7.7% for EZE10, and 7.7% for ATV10. In the very high‐risk category, achievement rates were 4.0% for EZE10/ATV5 and 8.3% for ATV10. Differences in achievement rates between the EZE10/ATV5 group and other groups were statistically significant primarily in the low to moderate or high‐risk categories.

### Subgroup analyses by CVD risk factors

In subgroup analyses, patients without diabetes showed a greater LDL‐C reduction (−47.3%) compared with those with diabetes (−38.0%) in the EZE10/ATV5 group (Figure S3A,B). In those with hypertension, the EZE10/ATV5 group resulted in a − 44.3% reduction, while patients without hypertension achieved −45.4% (Figure S4A,B). Among patients aged 65 years and older, LDL‐C reductions were − 45.9% for the EZE10/ATV5 group and − 49.4% for those aged 75 years and older (Figure S5A,B). The EZE10/ATV5 treatments consistently showed superior LDL‐C lowering effects compared with other treatments within each subgroup. The rate of achievement of LDL‐C goals was also higher, regardless of the presence of major factors, except in patients with diabetes, where no significant differences were observed (Figure S6A,B for hypertension status and S6C,D for diabetic status).

### Adverse events

Among the 233 participants included in the safety analysis, the incidence of AEs was lowest in the EZE10/ATV5 group, with only four participants (6.9%) reporting an AE. In comparison, AEs were reported in nine participants (15.0%) in the EZE10 group, seven participants (12.3%) in the ATV5 group, and 16 participants (27.6%) in the ATV10 group (Table 2). These differences between the treatment groups were statistically significant (*p* = 0.017). The most common AEs were headache (1.7%–3.5%), dizziness (1.7%–3.3%), nasopharyngitis (1.7%–3.5%), and dyspepsia (1.7%–1.8%). Musculoskeletal AEs, such as myalgia, arthralgia, and pain in extremities, were observed in three participants receiving atorvastatin (one each in the EZE10/ATV5, ATV5, and ATV10 groups). Most AEs were mild (6.9%–19.0%) or moderate (1.7%–8.6%) in severity, and no severe AEs were reported in any group. The rates of AE‐related study drug discontinuation were similar across groups (0%–3.5%, *p* = 0.756). In the ATV10 group, one participant experienced an increase in aspartate aminotransferase and alanine aminotransferase levels to more than three times the upper limit of normal. This was likely related to preexisting high baseline levels and obesity rather than the investigational drug. Additionally, no participant experienced an increase in creatinine kinase levels to more than 10 times the upper limit of normal. No clinically significant differences in laboratory results, physical signs, vital signs, or weight changes were observed between the treatment groups.

**TABLE 2:** Summary of adverse events.

	EZE10/ATV5 (*n* = 58)	EZE10 (*n* = 60)	ATV5 (*n* = 57)	ATV10 (*n* = 58)	*p*‐value
Adverse event (AE)^a^	4 (6.9) [5]	9 (15.0) [16]	7 (12.3) [13]	16 (27.6) [25]	0.017
Headache	1 (1.7) [1]	2 (3.3) [5]	2 (3.5) [2]	‐	0.756
Dizziness	1 (1.7) [1]	2 (3.3) [2]		1 (1.7) [1]	0.627
Nasopharyngitis	‐	‐	2 (3.5%) [2]	1 (1.7) [1]	0.324
Dyspepsia	‐	1 (1.7) [2]	1 (1.8) [1]	‐	0.589
Adverse drug reaction	1 (1.7) [2]	1 (1.7) [1]	1 (1.7) [3]	1 (1.7) [1]	1.00
Serious adverse event	‐	‐	‐	‐	‐
AE leading to study drug discontinuation	1 (1.7) [1]	1 (1.7) [1]	‐	2 (3.5) [3]	0.756
Severity of AE^b^					
Mild	4 (6.9) [4]	8 (13.3) [9]	6 (10.5) [7]	11 (19.0) [19]	0.954
Moderate	1 (1.7) [1]	3 (5.0) [7]	3 (5.3) [6]	5 (8.6) [6]	0.842
Severe	‐	‐	‐	‐	‐

*Note*: Data are presented as number of participants (%) [number of events].

Abbreviations: AE, adverse event; ATV5, atorvastatin 5 mg monotherapy; ATV10, atorvastatin 10 mg monotherapy; EZE10, ezetimibe 10 mg monotherapy; EZE10/ATV5, combination therapy of ezetimibe 10 mg and atorvastatin 5 mg.

^a^ Adverse events occurring during the clinical trial are categorized by preferred terms, and those considered to be significantly correlated by the clinical judgment are selected, which may result in the total number of events differing from the subcategories.

^b^ The total number of participants may differ due to multiple adverse events reported by a single participant.

## DISCUSSION

In this study, we evaluated the effect of low‐dose atorvastatin and ezetimibe combination therapy on LDL‐C levels over 8 weeks, compared with monotherapy of either atorvastatin or ezetimibe, and with monotherapy of moderate‐intensity atorvastatin. The main study findings can be summarized as follows: (1) the combination therapy resulted in a significant LDL‐C reduction of 44.8%, compared with 32.0% with ATV 10, and achieved a larger LDL reduction than that achieved by atorvastatin or ezetimibe monotherapies; (2) the combination therapy achieved an overall LDL‐C goal achievement rate of 41.1%, which was significantly higher than those of the monotherapy groups, especially in patients with low to moderate or high cardiovascular risk; and (3) the combination therapy also showed favorable effects on TC, non‐HDL‐C, and Apo B levels, with AEs being infrequent across all the groups.

Combination therapy, which involves adding non‐statin agents to statin therapy, is recommended for patients who do not achieve adequate LDL‐C reduction with statins alone or those who are at very high cardiovascular risk. This approach is more effective than only increasing the statin dosage. Studies have shown that adding 10 mg of ezetimibe to different doses of atorvastatin (10, 20, or 40 mg) is more likely to achieve LDL targets than using higher doses of atorvastatin alone (Conard et al., 2008; Uemura et al., 2012). However, despite the recognized efficacy of moderate‐ to high‐intensity statin–ezetimibe combinations, little is known about whether combining ezetimibe with even lower doses of atorvastatin (e.g., 5 mg) can yield clinically meaningful LDL‐C reduction. Our study notably addresses a gap in the literature. In our study, participants treated with ezetimibe and low‐dose atorvastatin (EZE10/ATV5) had greater LDL‐C reductions than those on ATV10 alone, demonstrating the 13–20% additional LDL‐C reduction typically seen when adding ezetimibe to statins (Morrone et al., 2012). Previous landmark trials established the efficacy of moderate‐intensity statin–ezetimibe combinations in high‐risk patients. In the Improved Reduction of Outcomes: Vytorin Efficacy International Trial (IMPROVE‐IT), simvastatin–ezetimibe offered significant cardiovascular benefits for individuals with acute coronary syndrome, while the Randomized Comparison of Moderate‐Intensity Statin Plus Ezetimibe Combination Therapy versus High‐Intensity Statin Therapy in Patients with Stable Coronary Artery Disease (RACING) trial demonstrated non‐inferior outcomes with rosuvastatin–ezetimibe compared with high‐intensity statin monotherapy (Cannon et al., 2015; Kim et al., 2022). Post hoc analyses confirmed these advantages across diverse risk groups, solidifying moderate‐intensity statin–ezetimibe therapy in routine practice. Our findings extend this approach by showing that even a lower‐dose atorvastatin regimen (5 mg) combined with ezetimibe (10 mg) can achieve substantial LDL‐C reductions in Korean patients with primary hypercholesterolemia, thereby broadening the statin–ezetimibe strategy to include lower‐dose regimens. This provides an effective alternative for patients requiring robust LDL‐C control who may not tolerate higher‐intensity statin therapy.

Current clinical practice guidelines recommend assessing cardiovascular disease (CVD) risk for atherosclerotic CVD prevention, tailoring the intensity of lipid‐lowering interventions to each patient's risk profile (Cooney et al., 2009; Cooney et al., 2010; Hajifathalian et al., 2015). Given these recommendations, our findings highlight the potential role of low‐dose statin–ezetimibe therapy as an alternative approach—especially for those at moderate to high risk of CVD who might experience side effects with higher‐intensity regimens. Although higher‐dose statins may exhibit more pronounced pleiotropic anti‐inflammatory effects (Davignon, 2004), meta‐analyses suggest that cardiovascular benefits predominantly arise from absolute LDL‐C reduction rather than these additional properties (Robinson et al., 2005). This finding further strengthens the rationale for our investigation of low‐dose statin‐ezetimibe combinations, which maximize LDL‐C reduction through complementary mechanisms. While guidelines recommend moderate‐ to high‐intensity statins for patients at moderate to high risk of CVD, our study demonstrates that low‐dose atorvastatin (5 mg) combined with ezetimibe (10 mg) achieves significantly greater LDL‐C reductions compared with either monotherapy across diverse patient subgroups, including those stratified by age, diabetes status, and hypertension. These findings suggest that low‐dose statin–ezetimibe therapy could be considered among the viable treatment options for Asian patients at elevated CVD risk who may not tolerate higher‐intensity statin doses.

In this study, EZE10/ATV5 significantly reduced TC, non‐HDL‐C, and Apo B levels—lipid profiles relevant to CVD prevention—and demonstrated an improved safety profile. The safety and tolerability observed were generally consistent with previous findings from ezetimibe and atorvastatin therapy of a similar duration (Bytyçi et al., 2022). Additionally, there were no serious AEs, and the incidence of hepatitis and liver, muscle, gastrointestinal, and allergic reactions was low. Notably, the EZE10/ATV5 group experienced significantly fewer AEs than those in the group treated with the usual dose of atorvastatin (ATV10). This suggests that coadministration of ezetimibe with low‐dose atorvastatin is a safer option for Korean patients with primary hypercholesterolemia, maintaining efficacy while minimizing side effects.

This study had several limitations. First, despite having adequate power, the relatively small sample size limits the generalizability of our results to broader patient populations. Second, the treatment duration was insufficient to comprehensively evaluate long‐term safety profiles and cardiovascular outcomes. Third, the study's adherence rates likely exceeded those typically observed in real‐world clinical settings. Fourth, we did not perform a cost‐effectiveness analysis, which could inform treatment decisions. Finally, as this study was conducted exclusively in Korean patients, the results cannot be directly applied to other Asian populations. Future research should address these limitations by investigating long‐term cardiovascular outcomes with low‐dose statin‐ezetimibe combination therapy across diverse Asian populations. Studies exploring cost‐effectiveness, optimal treatment duration, and the potential for dose adjustment in patients achieving sustained target LDL‐C levels would provide valuable clinical guidance. Additionally, examining this combination approach in secondary prevention settings could further establish its role as a tailored treatment option for patients with elevated CVD risk who may not tolerate higher statin doses.

## CONCLUSION

In this study, combining low‐dose atorvastatin (5 mg) with ezetimibe (10 mg) provided enhanced lipid‐lowering efficacy and a favorable safety profile compared with either agent alone in Korean patients with primary hypercholesterolemia. However, in high‐to very high‐risk patients, LDL‐C goal achievement remained limited (15.8% with combination therapy vs. 8.1% with atorvastatin 10 mg alone), highlighting the need for more intensive treatments in this subgroup. Nonetheless, the observed improvement with low‐dose statin–ezetimibe therapy suggests its potential value as an alternative option for individuals who cannot tolerate higher‐intensity statin regimens. These findings contribute to the growing evidence supporting the use of low‐dose statin–ezetimibe combinations within personalized dyslipidemia management strategies, particularly for patients who need effective LDL‐C reduction but cannot tolerate high‐intensity statin therapy.

### Tweet

Combination therapy with low‐dose atorvastatin and ezetimibe shows superior LDL‐C reduction and safety in Koreans, emerging as a promising option for primary hypercholesterolemia treatment. #LDL #Hypercholesterolemia #Atorvastatin #Ezetimibe.

## AUTHOR CONTRIBUTIONS

**Tae Oh Kim:** Investigation; data collection; visualization; and writing—original draft. **Kyounghoon Lee:** Formal analysis and investigation. **Jin‐Man Cho:** Project administration and investigation. **Hyuck‐Jun Yoon:** Investigation. **Tae‐Ho Park:** Investigation. **Jung Hyun Choi:** investigation. **Jung‐Won Suh:** Investigation. **Seok‐Yeon Kim:** Investigation. **Hong‐Seok Lim:** Investigation. **Jong‐Seon Park:** Investigation. **Deok‐Kyu Cho:** Investigation. **Gyung‐Min Park:** Investigation. **Sung‐Gyun Ahn:** Investigation. **Sanghoon Shin**: Investigation. **Sung Uk Kwon:** Investigation. **Dae‐Hyeok Kim:** Investigation. **Sang‐Rok Lee:** Investigation. **Jung‐Hoon Sung**: Investigation. **Hwan‐Cheol Park:** Investigation. **Seung‐Whan Lee:** Conceptualization; data curation; supervision; visualization; writing—original draft; and writing—review and editing.

## FUNDING INFORMATION

The Department of Clinical Development at Chong Kun Dang Pharmaceutical Corp. supported the supply of the investigational drug.

## CONFLICT OF INTEREST STATEMENT

The authors declare that they have no conflict of interest.

## ETHICS STATEMENT

The study was conducted in accordance with the ethical principles of the Declaration of Helsinki, adhering to the principles of Good Clinical Practice. The study protocol was approved by the Institutional Review Board (IRB) at each participating center (Asan Medical Center IRB number: 2022‐1302) and registered at www.clinicaltrials.gov (NCT05657574). Written informed consent was obtained from all participants before enrollment. All data assessment and processing were completed in compliance with applicable data protection regulations.

## Supporting information


**Data S1** Supporting information.

## Data Availability

The data that support the findings of this study are available from the corresponding author upon reasonable request.
