# Supplementary material for: Efficacy and safety of low‐dose atorvastatin plus ezetimibe for primary hypercholesterolemia: A randomized, double‐blind, multicenter phase 3 trial
Source: Lipids. 2025 Apr 18;60(5):317–26. doi: 10.1002/lipd.12442 (PMC12434550; doi:10.1002/lipd.12442)
Supplement: Supplementary file 1 — Data S1 Supporting information. [file LIPD-60-317-s001.docx]

**Supplementary Material**

This appendix is provided by the authors to provide readers with additional information about their work. Supplement to: TO Kim et al. "Efficacy and Safety of Combined Low-Dose Atorvastatin and Ezetimibe in Patients with Primary Hypercholesterolemia: A Randomized, Double-Blind, Multicenter, Therapeutic Confirmatory, Phase 3 Clinical Trial"

**Supplemetary Table S1.** LDL-C levels for enrollment and treatment goals by CVD risk category

**Supplemetary Table S2.** Treatment Compliance

**Supplementary Table S3.** Changes in lipid parameters at week 4 and week 8

**Supplementary Figure S1.** Study Design and Randomization

**Supplementary Figure S2.** Percentage Change in LDL-C Levels from Baseline at 4 and 8 Weeks

**Supplementary Figure S3.** LDL-C Changes by Treatment Group Based on Diabetes Status

**Supplementary Figure S4.** LDL-C Changes by Treatment Group Based on Hypertension Status

**Supplementary Figure S5.** LDL-C Changes by Treatment Group Based on Age

**Supplementary Figure S6.** LDL-C Goal Achievement by Patient Health Conditions

**Supplementary Table S1.** LDL-C levels for enrollment and treatment goals by CVD risk category

| **Risk Category** | | **Enrollment criteria** | | **LDL-C treatment goal** |
| --- | --- | --- | --- | --- |
|  |  | **LDL-C** | **TAG** |  |
| Very High* | +1 very high risk factor or  10-year risk^†^ ≥10% | 55–190 mg/dL | < 500 mg/dL | ≥ 50% reduction from baseline and  < 55 mg/dL |
| High* | +1 high risk factor or  5% ≤ 10-year risk^†^ <10% | 70–190 mg/dL |  | ≥ 50% reduction from baseline and  < 70 mg/dL |
| Moderate* | 1% ≤ 10-year risk^†^ <5% | 100–250 mg/dL |  | < 100 mg/dL |
| Low* | 10-year risk^†^ <1% | 116–250 mg/dL |  | < 116 mg/dL |
| *Very High Risk: Patients with documented ASCVD (e.g., previous ACS, stable angina, coronary revascularization, stroke, TIA, or peripheral arterial disease). This also includes those with DM and target organ damage or multiple major risk factors, severe CKD (eGFR <30 mL/min/1.73 m²), SCORE ≥10% for 10-year fatal CVD risk, or FH with ASCVD or another major risk factor.  *High Risk: Patients with markedly elevated single risk factors, such as total cholesterol >310 mg/dL, LDL-C >190 mg/dL, or blood pressure ≥180/110 mmHg. This group also includes patients with familial hypercholesterolemia without other major risk factors, patients with DM and duration >10 years or an additional risk factor, moderate CKD (eGFR 30–59 mL/min/1.73 m²), or a calculated SCORE between 5% and 10% for a 10-year risk of fatal CVD.  *Moderate Risk: Young patients (type 1 DM <35 years; type 2 DM <50 years) with diabetes duration <10 years without other risk factors, or a calculated SCORE between 1% and 5% for a 10-year risk of fatal CVD.  *Low Risk: Patients with a calculated SCORE <1% for a 10-year risk of fatal CVD.  ^†^The 10-year risk was calculated using the Systematic COronary Risk Evaluation2 (SCORE2) method, which includes factors such as sex, age, smoking status, systolic blood pressure, and total and HDL cholesterol levels. | | | | |

Abbreviations: ACS = acute coronary syndrome; ASCVD = atherosclerotic cardiovascular disease; CKD = chronic kidney disease; CVD = cardiovascular disease; DM = diabetes mellitus; eGFR = estimated glomerular filtration rate; FH = family history; LDL-C = low-density lipoprotein-cholesterol; SCORE = Systematic COronary Risk Evaluation; TC = total cholesterol; TAG = triacylglycerol; TIA = transient ischemic attack

**Supplementary Table S2.** Treatment Compliance

|  | **EZE10/ATV5 (n=59)** | **EZE10 (n=60)** | **ATV5 (n=58)** | **ATV10 (n=58)** | ***P*-value*** |
| --- | --- | --- | --- | --- | --- |
| Mean (SD) | 97.5 (13.9) | 97.7 (5.4) | 95.2 (13.9) | 96.4 (10.0) | 0.324 |
| Median | 100.0 | 100.0 | 98.3 | 100.0 |  |
| Min, Max | 0.0, 124.1 | 68.8, 101.9 | 0.0, 105.8 | 28.6, 107.1 |  |

Treatment compliance (%) = (number of medications actually taken / number of medications that should be taken in a given period) × 100.

During the treatment period (visit 2 to visit 4), adherence was assessed for each treatment group, with results expressed as mean ± SD or as n (%).

Abbreviations: ATV5 = atorvastatin 5 mg monotherapy; ATV10 = atorvastatin 10 mg monotherapy; EZE10 = ezetimibe 10 mg monotherapy; EZE10/ATV5 = combination therapy of ezetimibe 10 mg and atorvastatin 5 mg.

**Supplementary Table S3.** Changes in lipid parameters at week 4 and week 8

|  | **EZE10/ATV5 (n=56)** | | **EZE10 (n=56)** | | **ATV5 (n=55)** | | **ATV10 (n=55)** | |
| --- | --- | --- | --- | --- | --- | --- | --- | --- |
|  | **Mean(SD)** | ***P*-value*** | **Mean(SD)** | ***P*-value*** | **Mean(SD)** | ***P*-value*** | **Mean(SD)** | ***P*-value*** |
| **LDL-C (mg/dL)** |  |  |  |  |  |  |  |  |
| Baseline | 143.8 (25.8) |  | 137.4 (26.9) |  | 137.3 (25.0) |  | 133.8 (27.4) |  |
| Visit 3 (4 weeks) | 78.2 (22.2) |  | 115.1 (25.2) |  | 100.7 (23.0) |  | 84.9 (21.2) |  |
| Visit 4 (8 weeks) | 78.6 (20.8) |  | 116.9 (24.6) |  | 98.4 (22.2) |  | 88.0 (20.2) |  |
| CFB at Visit 3 (4 week) | -45.1 (15.3) | <0.0001 | -15.1 (17.1) | <0.0001 | -25.5 (17.6) | <0.0001 | -34.7 (19.0) | <0.0001 |
| CFB at Visit 4 (8 week) | -44.8 (13.5) | <0.0001 | -12.7 (21.9) | <0.0001 | -27.3 (15.7) | <0.0001 | -32.1 (19.7) | <0.0001 |
| **TC (mg/dL)** |  |  |  |  |  |  |  |  |
| Baseline | 216.6 (33.0) |  | 211.8 (32.7) |  | 209.2 (30.5) |  | 205.4 (29.7) |  |
| Visit 3 (4 weeks) | 148.0 (26.3) |  | 188.2 (28.7) |  | 170.3 (25.0) |  | 155.2 (23.8) |  |
| Visit 4 (8 weeks) | 150.3 (27.1) |  | 188.6 (26.7) |  | 169.6 (25.6) |  | 156.8 (23.7) |  |
| CFB at Visit 3 (4 weeks) | -31.2 (11.1) | <0.0001 | -10.4 (11.8) | <0.0001 | -17.8 (11.9) | <0.0001 | -23.6 (12.7) | <0.0001 |
| CFB at Visit 4 (8 weeks) | -30.2 (10.9) | <0.0001 | -9.9 (13.3) | <0.0001 | -18.2 (11.2) | <0.0001 | -22.8 (13.1) | <0.0001 |
| **Non HDL-C (mg/dL)** |  |  |  |  |  |  |  |  |
| Baseline  Visit 3 (4 weeks)  Visit 4 (8 weeks) | 165.9 (29.7) |  | 160.2 (32.9) |  | 160.8 (28.5) |  | 155.8 (28.1) |  |
|  | 95.2 (23.0) |  | 136.7 (29.8) |  | 118.6 (26.9) |  | 103.5 (23.8) |  |
|  | 99.2 (26.1) |  | 135.8 (29.1) |  | 117.7 (26.7) |  | 104.3 (21.9) |  |
| CFB at Visit 3 (4 weeks) | -41.9 (14.1) | <0.0001 | -13.8 (14.1) | <0.0001 | -25.8 (15.2) | <0.0001^W^ | -32.7 (14.9) | <0.0001 |
| CFB at Visit 4 (8 weeks) | -39.8 (14.5) | <0.0001 | -13.8 (16.8) | <0.0001 | -26.1 (14.1) | <0.0001^T^ | -31.9 (15.3) | <0.0001 |
| **Apo B (mg/dL)** |  |  |  |  |  |  |  |  |
| Baseline | 119.8 (20.6) |  | 116.3 (22.6) |  | 119.0 (23.1) |  | 115.5 (21.6) |  |
| Visit 3 (4 weeks) | 77.0 (17.5) |  | 104.5 (21.8) |  | 92.4 (20.2) |  | 83.6 (15.9) |  |
| Visit 4 (8 weeks) | 79.0 (17.7) |  | 103.9 (21.4) |  | 92.4 (20.7) |  | 83.5 (15.3) |  |
| CFB at Visit 3 (4 weeks) | -35.2 (13.2) | <0.0001 | -9.4 (13.3) | <0.0001 | -21.5 (15.2) | <0.0001^W^ | -26.3 (15.1) | <0.0001 |
| CFB at Visit 4 (8 weeks) | -33.7 (12.2) | <0.0001 | -9.4 (16.2) | <0.0001 | -21.6 (14.0) | <0.0001^W^ | -25.9 (17.7) | <0.0001 |
| **TAG (mg/dL)** |  |  |  |  |  |  |  |  |
| Baseline | 158.7 (79.5) |  | 169.5 (103.1) |  | 169.6 (92.5) |  | 154.5 (63.1) |  |
| Visit 3 (4 weeks) | 119.5 (50.9) |  | 160.3 (115.3) |  | 132.3 (58.7) |  | 123.3 (53.5) |  |
| Visit 4 (8 weeks) | 148.4 (140.7) |  | 145.0 (97.7) |  | 135.1 (81.0) |  | 116.8 (39.6) |  |
| CFB at Visit 3 (4 weeks) | -16.8 (30.9) | <0.0001 | -0.1 (37.6) | 0.406 | -13.2 (31.8) | 0.0004^W^ | -15.2 (35.6) | <0.0001^W^ |
| CFB at Visit 4 (8 weeks) | -2.9 (94.9) | 0.001 | -6.9 (32.2) | 0.054 | -13.5 (34.2) | 0.0001^W^ | -17.4 (31.3) | <0.0001^W^ |
| **HDL-C (mg/dL)** |  |  |  |  |  |  |  |  |
| Baseline | 50.7 (14.1) |  | 51.7 (13.2) |  | 48.4 (14.5) |  | 49.6 (11.7) |  |
| Visit 3 (4 weeks) | 52.7 (13.0) |  | 51.5 (13.3) |  | 51.7 (13.9) |  | 51.7 (13.2) |  |
| Visit 4 (8 weeks) | 51.1 (13.0) |  | 52.8 (13.1) |  | 51.8 (14.3) |  | 52.5 (14.1) |  |
| CFB at Visit 3 (4 weeks) | 5.6 (14.1) | 0.015 | 0.6 (13.2) | 0.733 | 8.2 (11.9) | <0.0001 | 4.8 (13.1) | 0.009 |
| CFB at Visit 4 (8 weeks) | 2.3 (15.8) | 0.612 | 3.1 (12.3) | 0.063 | 8.6 (14.4) | <0.0001 | 6.3 (16.1) | 0.005 |
| **Apo A1 (mg/dL)** |  |  |  |  |  |  |  |  |
| Baseline | 150.2 (26.2) |  | 151.5 (23.8) |  | 144.6 (26.4) |  | 151.2 (23.8) |  |
| Visit 3 (4 weeks) | 156.9 (25.8) |  | 154.6 (26.9) |  | 154.0 (28.7) |  | 159.1 (28.6) |  |
| Visit 4 (8 weeks) | 156.8 (25.1) |  | 156.0 (24.4) |  | 154.5 (27.4) |  | 160.9 (31.1) |  |
| CFB at Visit 3 (4 weeks) | 5.0 (9.5) | 0.0002 | 2.6 (12.6) | 0.127 | 6.9 (11.4) | <0.0001 | 5.2 (9.3) | 0.0001 |
| CFB at Visit 4 (8 weeks) | 5.3 (11.9) | 0.002 | 3.7 (11.3) | 0.018 | 7.6 (12.1) | <0.0001 | 6.5 (12.1) | 0.0002 |
|  | | | | | | | | |
| *Paired T-test (T) or Wilcoxon Signed Rank Test (W) results of percentage changes to baseline | | | | | | | | |

Abbreviations: ATV5 = atorvastatin 5 mg monotherapy; ATV10 = atorvastatin 10 mg monotherapy; CFB = change from baseline; EZE10 = ezetimibe 10 mg monotherapy; EZE10/ATV5 = combination therapy of ezetimibe 10 mg and atorvastatin 5 mg; SD = standard deviation. TAG = triacyloglycerol; TC = total cholesterol.

**Supplementary Figure S1.** Study Design and Randomization


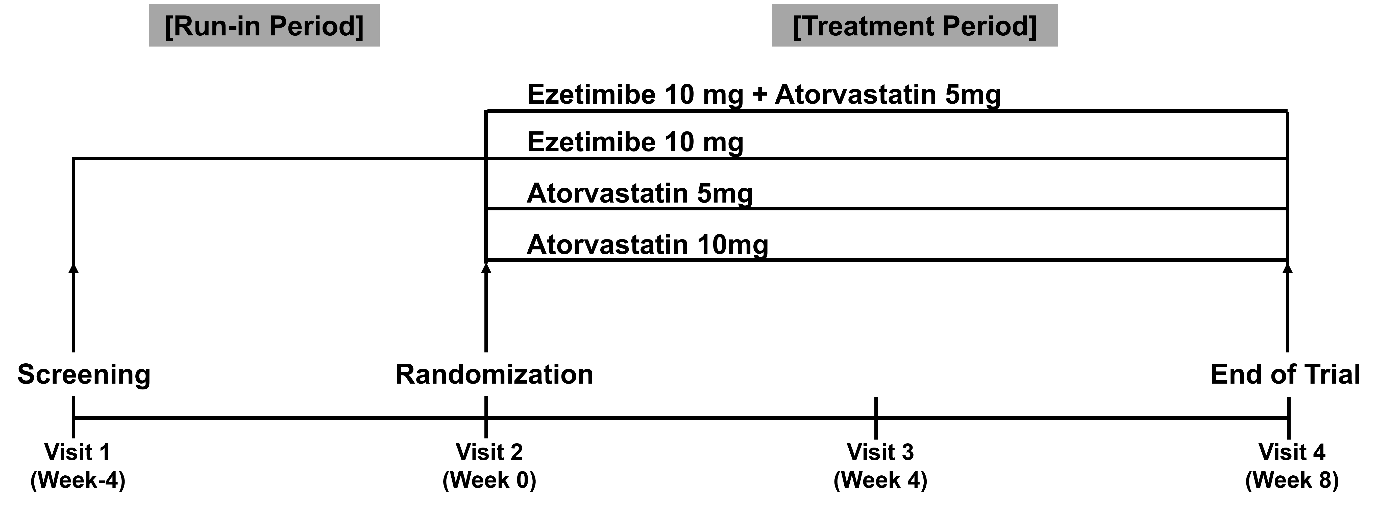


The study included a 4-week placebo run-in period where previous lipid-lowering drugs were discontinued, followed by an 8-week double-blind treatment period with participants randomized into four groups: Ezetimibe 10 mg and Atorvastatin 5 mg (EZE10/ATV5), Ezetimibe 10 mg (EZE10), Atorvastatin 5 mg (ATV5), and Atorvastatin 10 mg (ATV10). Randomization was stratified according to cardiovascular risk categories, and participants followed therapeutic lifestyle changes (TLC) during both periods.

**Supplementary Figure S2.** Percentage Change in LDL-C Levels from Baseline at 4 and 8 Weeks
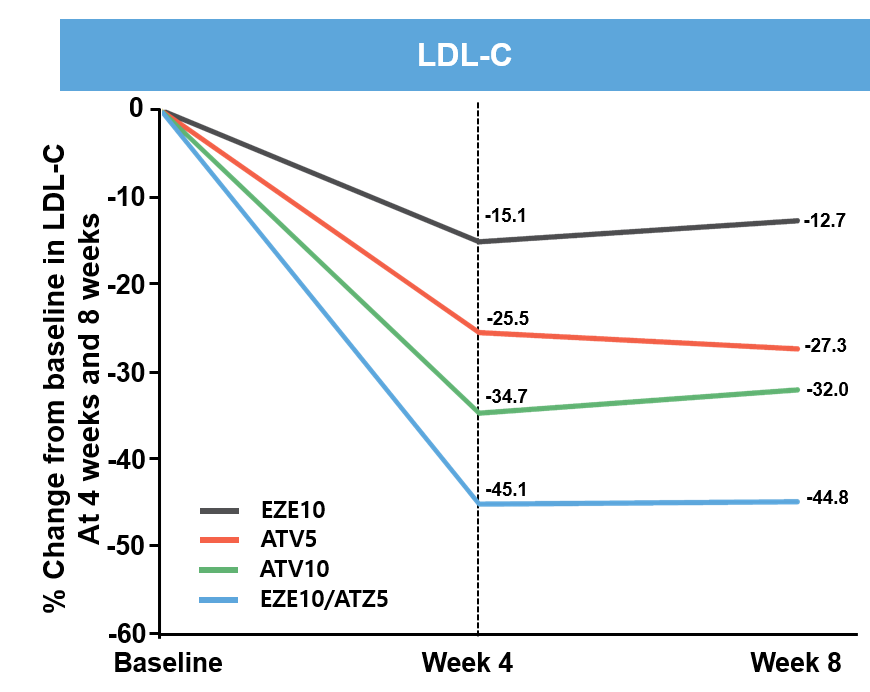


This figure shows the percentage change in LDL-C levels from baseline at 4 and 8 weeks for the treatment groups: EZE10, ATV5, ATV10, and EZE10/ATV5. Data are presented as mean. The EZE10/ATV5 group exhibited the largest LDL-C reduction.

Abbreviations: ATV5 = atorvastatin 5 mg monotherapy; ATV10 = atorvastatin 10 mg monotherapy; EZE10 = ezetimibe 10 mg monotherapy; EZE10/ATV5 = combination therapy of ezetimibe 10 mg and atorvastatin 5 mg.

**Supplementary Figure S3.** LDL-C Changes by Treatment Group Based on Diabetes Status


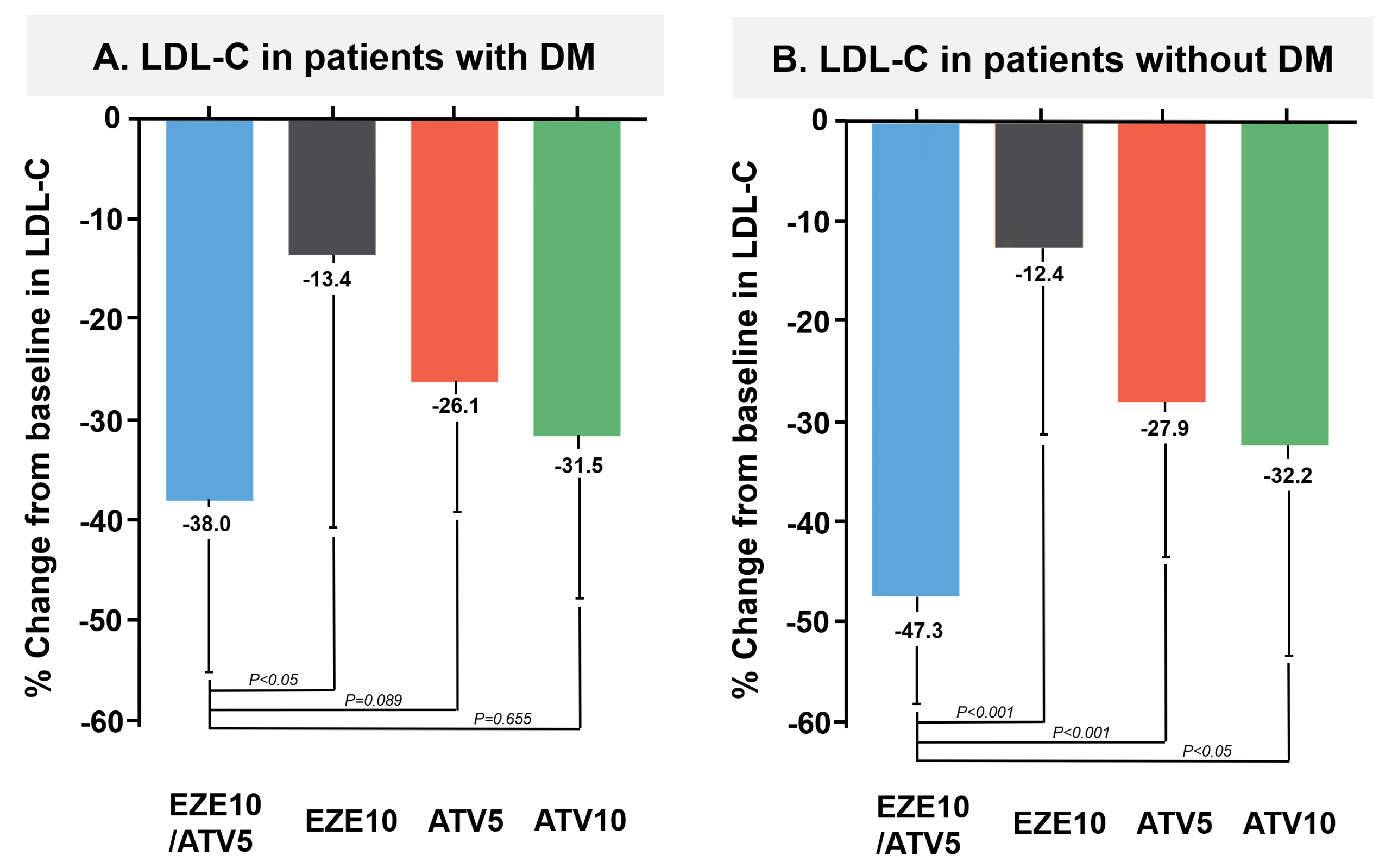


This figure shows the percentage change in LDL-C levels from baseline at 8 weeks for the treatment groups, stratified by diabetes status. Data are presented as mean ± standard deviation. The EZE10/ATV5 group exhibited the largest LDL-C reduction in both diabetic and non-diabetic subgroups.

Abbreviations: ATV5 = atorvastatin 5 mg monotherapy; ATV10 = atorvastatin 10 mg monotherapy; EZE10 = ezetimibe 10 mg monotherapy; EZE10/ATV5 = combination therapy of ezetimibe 10 mg and atorvastatin 5 mg; DM = diabetes mellitus.

**Supplementary Figure S4.** LDL-C Changes by Treatment Group Based on Hypertension Status
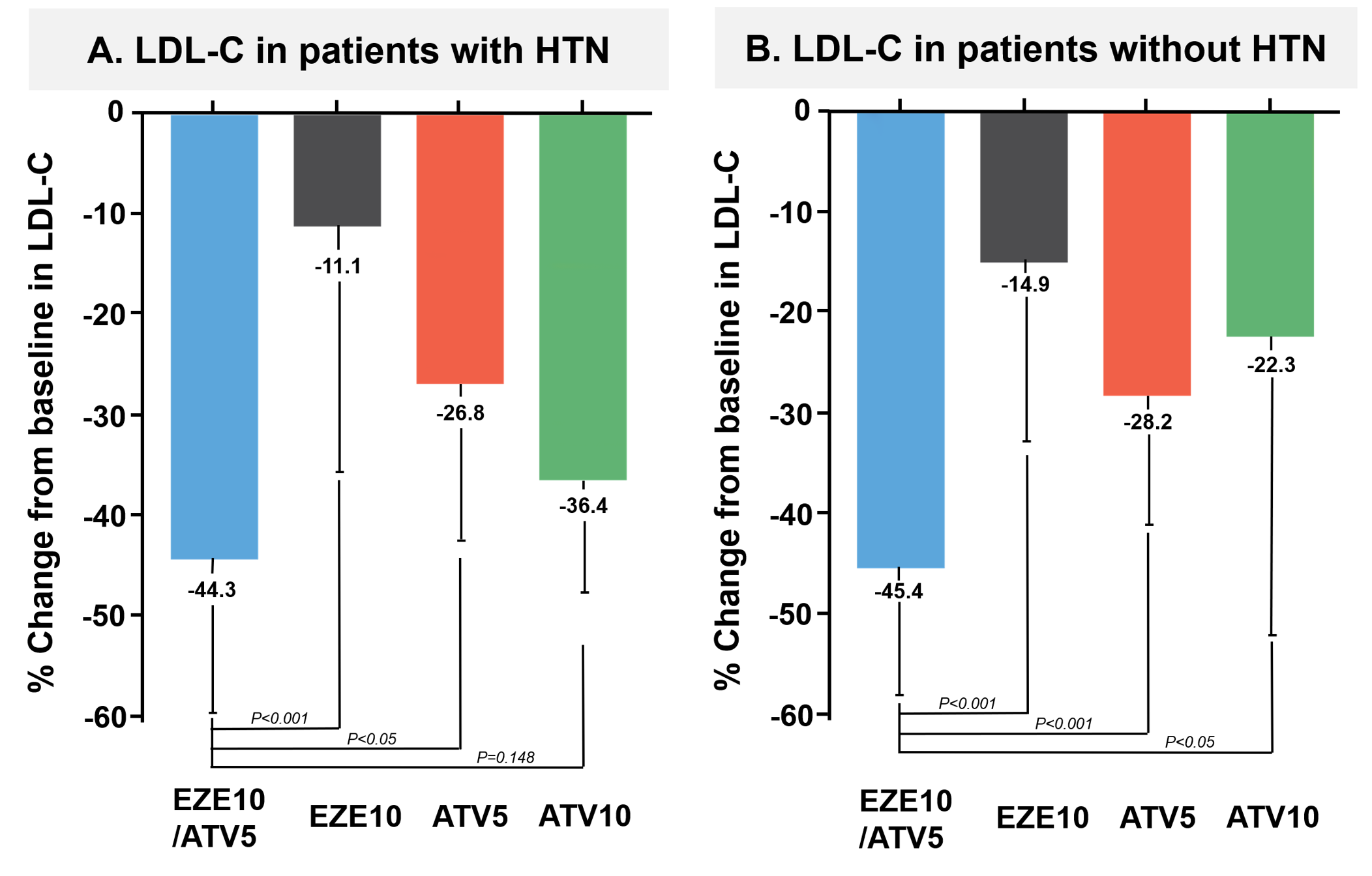


This figure shows the percentage change in LDL-C levels from baseline at 8 weeks for the treatment groups, stratified by hypertension status. Data are presented as mean ± standard deviation. The EZE10/ATV5 group exhibited the largest LDL-C reduction in both hypertensive and non-hypertensive subgroups.

Abbreviations: ATV5 = atorvastatin 5 mg monotherapy; ATV10 = atorvastatin 10 mg monotherapy; EZE10 = ezetimibe 10 mg monotherapy; EZE10/ATV5 = combination therapy of ezetimibe 10 mg and atorvastatin 5 mg; HTN = hypertension.

**Supplementary Figure S5.** LDL-C Changes by Treatment Group Based on Age


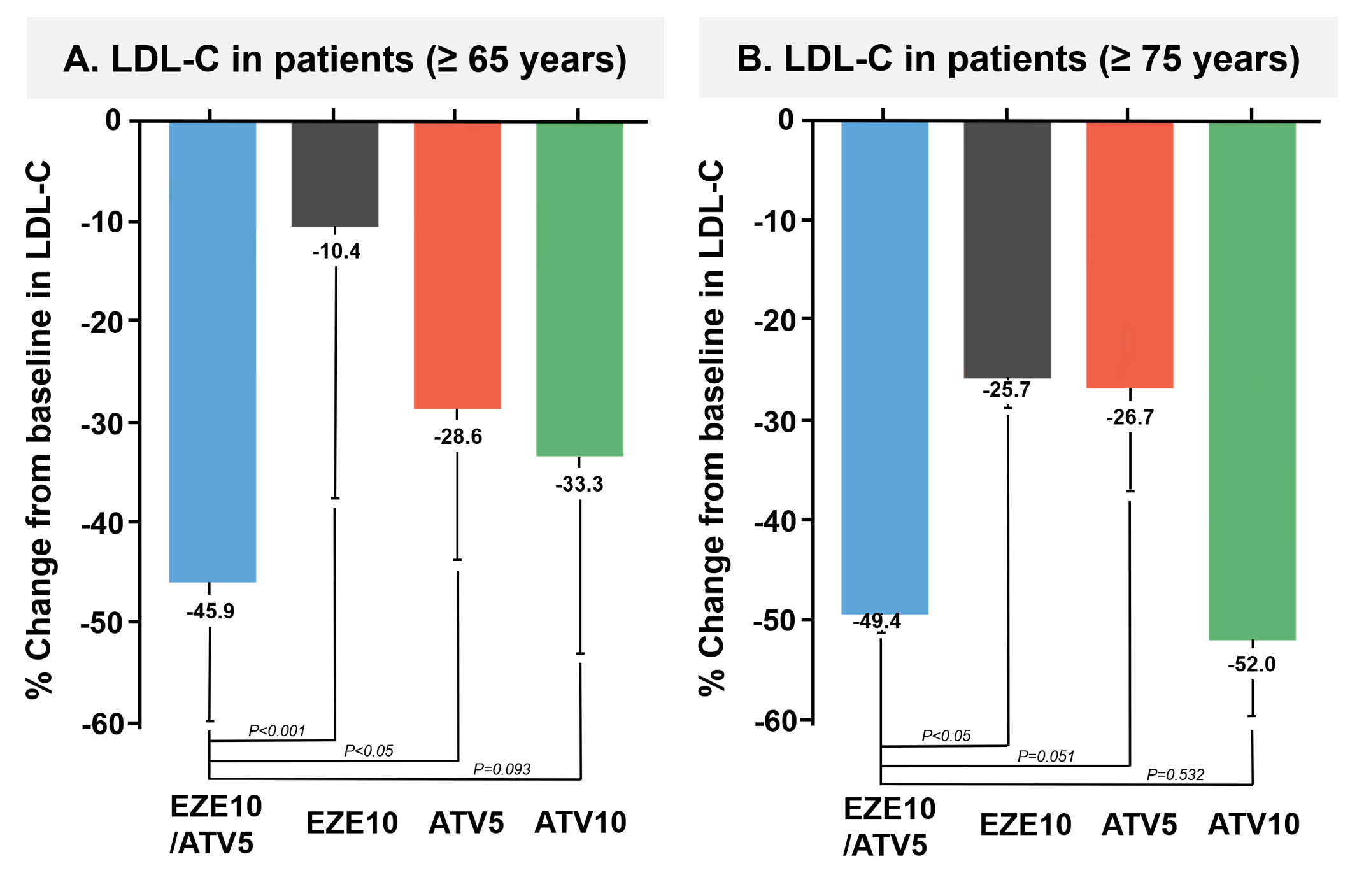


This figure shows the percentage change in LDL-C levels from baseline at 8 weeks for the treatment groups, stratified by age groups (≥65 years and ≥75 years). Data are presented as mean ± standard deviation. The EZE10/ATV5 group exhibited the largest LDL-C reduction across all age groups.

Abbreviations: ATV5 = atorvastatin 5 mg monotherapy; ATV10 = atorvastatin 10 mg monotherapy; EZE10 = ezetimibe 10 mg monotherapy; EZE10/ATV5 = combination therapy of ezetimibe 10 mg and atorvastatin 5 mg.

**Supplementary Figure S6. LDL-C Goal Achievement by Patient Health Conditions**

**
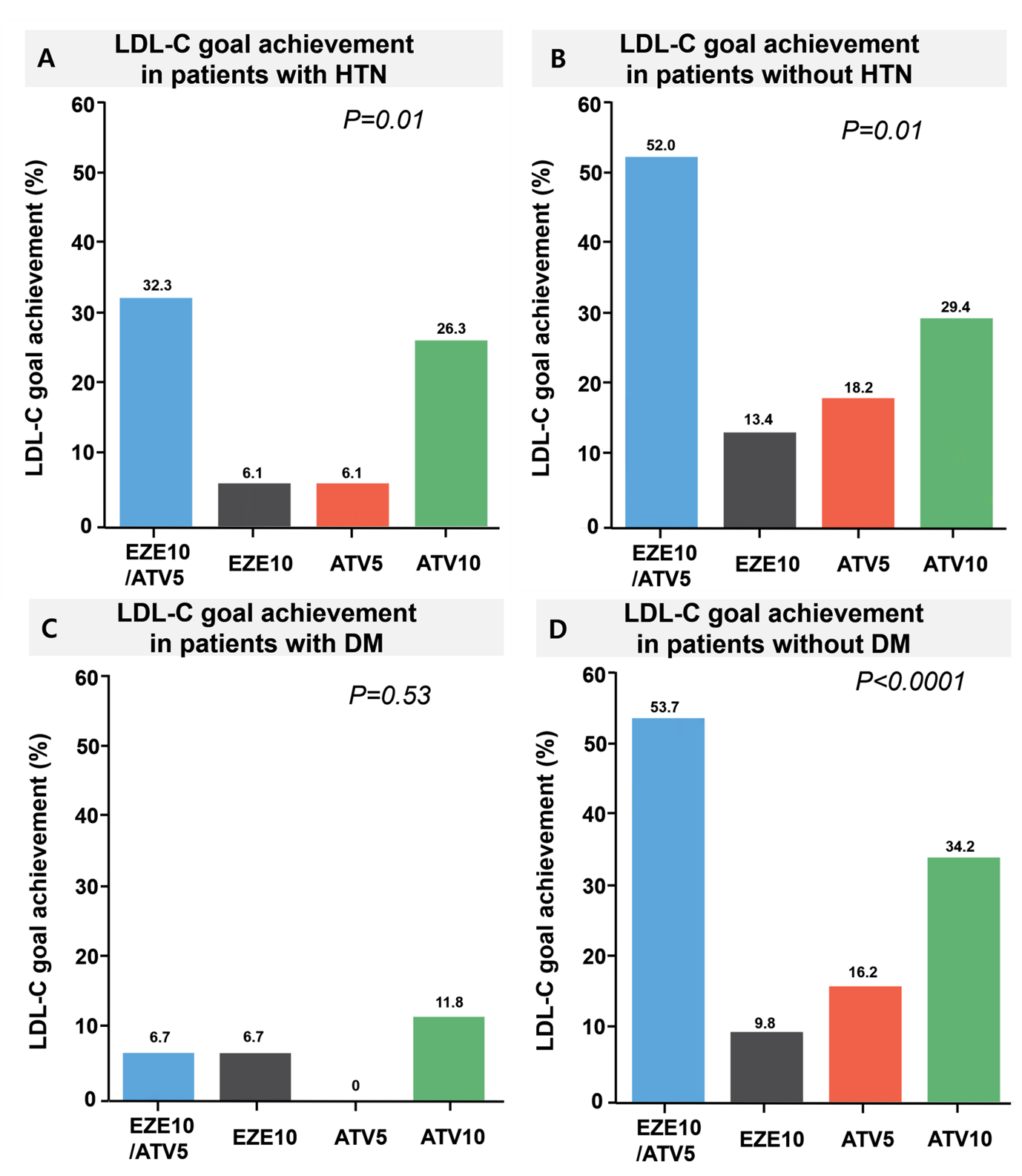
**

This figure shows the percentage of patients achieving risk-based LDL-C goals at 8 weeks for the treatment groups, stratified by the presence of hypertension and diabetes. The EZE10/ATV5 group exhibited the highest achievement of LDL-C goals in most subgroups, except in patients with diabetes where no significant differences were observed between treatment groups.

Abbreviations: ATV5 = atorvastatin 5 mg monotherapy; ATV10 = atorvastatin 10 mg monotherapy; EZE10 = ezetimibe 10 mg monotherapy; EZE10/ATV5 = combination therapy of ezetimibe 10 mg and atorvastatin 5 mg; DM = diabetes mellitus; HTN = hypertension.
